# The Female Advantage in Verbal Memory and its Relationship to Cognitive Status and Cortical Thickness, Brain Amyloid and Tau in Aging

**DOI:** 10.1002/alz70856_106047

**Published:** 2026-01-09

**Authors:** Hannah E Dewhurst, Nirupama Natarajan, Marissa A. Gogniat, Oscar L Lopez, Ann D Cohen, Beth E. Snitz

**Affiliations:** ^1^ University of Pittsburgh School of Medicine, Pittsburgh, PA, USA; ^2^ University of Pittsburgh, Pittsburgh, PA, USA; ^3^ University of Pittsburgh Alzheimer's Disease Research Center (ADRC), Pittsburgh, PA, USA

## Abstract

**Background:**

Recent evidence suggests there are sex differences in verbal memory with most evidence supporting a female advantage in cognitively unimpaired older adults, while findings in females with mild cognitive impairment (MCI) are more inconsistent. Paradoxically, females are disproportionately affected by Alzheimer's disease (AD). Sex differences in amyloid and tau burden and volumetric brain changes that proceed cognitive decline may help explain sex differences in AD risk. Examining sex as a moderating factor of associations between brain biomarkers and verbal memory could give insight into pathways of AD sex differences.

**Method:**

In Study 1, we examined differences in baseline verbal memory (CVLT long delayed recall) in a sample of older adults without dementia (*N* = 342, 51.8% Female), adjusting for age (M=78, SD=9.01), race (83% White) and education (M=15 years, SD=2.7), and stratifying by adjudicated cognitive status (cognitively unimpaired individuals (CU), *n* = 231 and MCI, *n* = 111). In Study 2, a sub‐sample of participants (*n* = 172) completed 3T MRI, PiB‐PET (amyloid), and AV‐1451‐PET (tau) imaging at follow‐up, quantifying an AD‐associated cortical thickness (CT) composite region, global amyloid burden, and tau burden in the medial temporal (MTL) and meta‐temporal (MT) regions. Sex stratified regression models examined relationships between biomarkers and concurrent delayed recall scores, followed by moderation models testing for a sex*biomarker interaction on delayed recall.

**Result:**

Study 1: In adjusted ANOVA models, delayed recall was higher in females (M=10.3, SD=2.6) than males (M=9.5, SD=2.9) in CU (*p* = 0.01) but not MCI (females: M=7.1, SD=3.6; males: M=6.1, SD=3.3). Study 2: In sex stratified regression models adjusted for age, education, and race, CT was the only biomarker associated with CVLT DR and only in females (B=6.45, *p* = 0.01), not males (B=‐3.01, *p* = 0.34), and not in non‐stratified models. In moderation models, the CT*sex interaction trended towards significant (*p* = 0.08). There was no sex*biomarker interaction for global amyloid or tau in MTL and MT regions.

**Conclusion:**

Female advantage in verbal memory was found in unimpaired but not older adults with MCI, consistent with female vulnerability to disease progression. Sex differences were not observed in the relationship between AD biomarkers and verbal memory, but results suggest cortical thickness may reflect female vulnerability to neurodegeneration more generally.

